# Topical or oral fluralaner efficacy against flea (*Ctenocephalides felis*) transmission of *Dipylidium caninum* infection to dogs

**DOI:** 10.1186/s13071-018-3140-x

**Published:** 2018-10-25

**Authors:** Deepa Gopinath, Leon Meyer, Jehane Smith, Rob Armstrong

**Affiliations:** 1 MSD Animal Health, 26 Talavera Rd, Macquarie Park, NSW 2113 Australia; 2 Clinvet International, Uitzich Road, Bainsvlei, Bloemfontein, 9338 South Africa; 3 Merck Animal Health, 2 Giralda Farms, Madison, NJ 07940 USA

**Keywords:** *Dipylidium caninum*, Dogs, Fluralaner, Flea control, Flea-borne disease

## Abstract

**Background:**

*Dipylidium caninum* is a common tapeworm of dogs contracted from ingestion of fleas containing the infective cysticercoid stage. Fluralaner is a systemically distributed isoxazoline class insecticide that delivers highly effective activity against fleas and ticks for up to 12 weeks after a single oral or topical treatment. This study evaluated the impact of this flea insecticidal efficacy on the transmission of *D. caninum* to dogs.

**Methods:**

Dogs were weighed and treated with a cestocide and then randomly assigned to 3 groups of 8. Fluralaner was administered topically (at the commercial dose) to one group and orally to another group while the third received topically administered sterile water. All dogs were subsequently infested with about 100 *D*. *caninum* infected *Ctenocephalides felis* at 7, 14, 21, 28, 35, 42, 49, 56, 63, 70, 77 and 83 days after treatment. Visual proglottid inspections and counts were conducted daily from 35 to 113 days post-treatment. Post-treatment *D. caninum* incidence was calculated for each group and compared between treated and untreated groups.

**Results:**

All 8 dogs in the placebo-treated group became infected with *D*. *caninum* while no shed proglottids were observed at any point during the post-treatment period from any dog in either fluralaner treated group.

**Conclusions:**

The insecticidal efficacy of a single treatment of either orally or topically administered fluralaner prevented *D. caninum* transmission from infected fleas to susceptible dogs for up to 12 weeks following administration.

## Background

*Dipylidium caninum*, commonly known as the flea tapeworm, is a frequently diagnosed intestinal cestode parasite of dogs and cats, although occasionally humans have become infected following ingestion of the saliva of infected pets [1]. *Ctenocephalides* spp. fleas, of which *C*. *felis* is the most prevalent on domestic dogs and cats [2, 3], are intermediate hosts in the life-cycle of *D*. *caninum*. In brief, *D*. *caninum* eggs passed in the feces of infected animals are ingested by flea larvae in the environment and the flea larvae then develop into pupae while hosting tapeworm embryos. The adult flea then emerges and infests a host, and within 2–3 days the hexacanth cestode embryo develops into an infective cysticercoid stage within the flea. The cysticercoid larvae require at least 24–36 hours before they are infective for the definitive host [4–6], a development period that is temperature-dependent [5]. Dogs and cats become infected when they ingest fleas containing the infective cysticercoid larvae during grooming. Adult *D*. *caninum* develop in the small intestine and within 2–3 weeks begin to shed egg packets called proglottids [7]. The overall pre-patent period can be as short as two weeks [6].

Clinical signs in *D*. *caninum* infected dogs generally consist of mild gastrointestinal manifestations and anal pruritus, which may cause the animal to exhibit ‘scooting’ behavior [8]. In addition, slowly motile proglottids may be observed on the animals’ feces and the combination of this behavior and the sight of proglottids can be distressing for owners [9]. The potential zoonotic transmission of this parasite [6, 10, 11] and wide geographical range [12] underline the value of protecting dogs and cats from *D*. *caninum*. Routine cestocidal treatment of household dog(s) and cat(s) is one option for managing this tapeworm [9]; however, the brief pre-patent period means that exposure to infective stages between cestocidal treatments can lead to infection and development of adult tapeworms in dogs. Dog owners easily underestimate the required frequency of cestocidal re-treatment administration needed to prevent *D*. *caninum* infections from reaching the egg shedding stage. Re-infection of pets can occur very quickly following cestocidal treatment, which has no residual effect [9].

Effective and persistent flea control can control the environmental proglottid load and prevent *D*. *caninum* infection, provided that fleas are killed sufficiently quickly before animals with fleas become tapeworm infected [4]. This is an additional benefit from an effective flea control regimen, adding to the control of other flea-related disorders such as flea-bite dermatitis and flea hypersensitivity dermatitis [2, 9]. PCR assessment found that 2.2% of *C*. *felis* from client-owned cats and 5.2% of *C*. *felis* from client-owned dogs in Europe were *D*. *caninum* infected [7]. These results show that the prevalence of *D*. *caninum* in Europe is sufficient to ensure that appropriate measures are routinely needed to prevent this tapeworm infection of domestic dogs and cats.

Fluralaner (Bravecto Chews and Bravecto Spot-On, Merck Animal Health, Madison, NJ, USA) is a highly effective flea insecticide that is systemically distributed in dogs following either topical or oral administration [13–15]. This active ingredient kills fleas following ingestion of a blood meal, with an onset of activity within two hours of initial oral administration [13]. Flea insecticidal efficacy following oral fluralaner administration reaches 98–100% at 8–24 hours after flea infestation [13] and efficacy of ≥ 99% has been demonstrated for 12 weeks following application of a single topical dose of fluralaner [13, 16, 17]. The hypothesis in this study is that fluralaner treatment will provide flea insecticidal efficacy that is sufficiently rapid to prevent *D*. *caninum* transmission to dogs infested with infected fleas. This result was previously reported in dogs treated orally with a combination of another isoxazoline class molecule, afoxolaner, and milbemycin against challenges with infected fleas over a 28-day study period [18]. The current study evaluated both orally and topically administered fluralaner at the recommended clinical dose (25–56 mg/kg) with flea challenges over the 12 week period following a single treatment. Fluralaner does not have a label indication against cestodes.

## Methods

This study was a parallel group designed, blinded, randomized, single center, placebo controlled efficacy study [19]. The study consisted of 24 dogs within 3 groups of 8 dogs each from an initial enrolled group of 28 dogs. Dogs were either beagles or mongrels (mixed-breed), and body weight ranged between 12.0–27.6 kg, with a mean body weight of 17.7 kg prior to commencement of the study (Day -3). Mean body weight within the groups was 17.7 kg in Group 1, 17.1 kg in Group 2 and 17.6 kg in Group 3. No statistically significant differences were recorded with respect to the body weights (*P* = 0.9640) measured in different groups, indicating homogeneity at the time of inclusion. There were four male and four female dogs within each group ranging from 12 to 85 months-old at the time of inclusion. To be included in the study, dogs had to have been clinically healthy on physical examination by a veterinarian on Day -7, older than 6 months of age at the time of inclusion, not clinically pregnant and not of an excessively fractious temperament which makes handling overtly difficult. The four dogs with the lowest body measurements on Day -2 were excluded from the study. Dogs included in the study had not been treated with a long-acting acaricide/insecticide during the 12 weeks preceding Day 0, and were also not treated with a macrocyclic lactone or other long acting anthelmintic during the three weeks preceding Day 0 (with the exception of deworming with a short-acting anthelmintic (a combination of praziquantel, pyrantel pamoate and febantel) during the study preparation phase prior to Day -7). None of the dogs were removed from the study prior to scheduled study termination and after inclusion on Day -3 (with the exception of the dogs that were infected with *D. caninum*).

Study dogs were acclimatized to conditions for 21 days before treatment and a centrifuged fecal parasite examination was run on all dogs on the first day of acclimatization to ensure the dogs were free of resident enteric parasites. The centrifuged fecal examination was conducted by thoroughly mixing the entire fecal sample of each dog to ensure a homogenized sample after collection. One gram of the homogenized sample was mixed with 10 ml of sugar solution and strained through a double layer of gauze. A 15 ml centrifuge tube was filled with the suspension and placed into the centrifuge and the tube was filled with sugar solution to a slight positive meniscus. A coverslip was placed on top of each tube, simultaneously ensuring that a small bubble was present under the coverslip. Samples were centrifuged in a swinging-head centrifuge at 1250× *rpm* for 5 min. After centrifugation, the tubes were removed and placed in a test tube rack and let stand for 10 min, then coverslips were removed and examined. All dogs were weighed and treated with a cestocide, a combination of milbemycin oxime and praziquantel (Milbemax®, Elanco, Greenfield, IN, USA). Their cages were screened daily for proglottids over the following 20-day acclimation period to detect any resident tapeworm infections persisting despite treatment.

Two days before treatment administration, dogs were ranked within sex in descending order of individual body weights, and blocked into 3 groups of 8 dogs each. One group was treated topically with sterile water, another group received orally administered fluralaner and the third received topically administered fluralaner. Fluralaner was dosed according to the product label, at a dose of 25–56 mg/kg body weight. Dogs treated with oral fluralaner were also treated with topical sterile water to maintain blinding. On treatment day, all dogs received half of their daily food ration approximately 20 min before treatment and the second half directly after treatment. All dogs were observed hourly for 6 h after treatment administration.

Thirty fleas per batch were sampled from at least three batches of fleas and examined microscopically for the presence of *D. caninum* metacestodes (Table 1) to determine the proportion containing the infective stage [20]. Approximately 100 *D*. *caninum*-infected *C. felis* fleas were placed on every dog in the study at 7, 14, 21, 28, 35, 42, 49, 56, 63, 70, 77 and 83 days after treatment.

**Table 1:** *Dipylidium caninum* infection in flea batches used to infest dogs using a natural challenge model

Time post-treatment (days)	Flea age (days)	Metacestode infection prevalence (%)^a^	Metacestode intensity per infected flea^b^	Metacestode abundance^c^
7	12	23.3	3.1	0.7
14	14	66.7	8.8	5.8
21	13	33.3	3.3	1.1
28	18	40.0	2.8	1.1
35	14	13.3	6.8	0.9
42	14	13.3	5.0	0.7
49	14	16.7	2.0	0.3
56	12	13.3	1.0	0.1
63	14	13.3	1.3	0.2
70	14	26.7	5.1	1.4
77	16	13.3	9.3	1.2
84	13	13.3	3.5	0.5

^a^No. infected fleas / total no. fleas examined

^b^Total no. metacestodes recovered / no. infected fleas

^c^Total no. metacestodes recovered / total no. of fleas examined

Visual proglottid inspections and counts were conducted daily on cage floors, sleeping areas and dog feces of all dogs from 35 to 113 days post-treatment to detect cestode-infected dogs. All dogs observed to have shed proglottids, and all dogs at the end of the study period, were removed from the study, dewormed and treated with a flea adulticide.

The experimental unit was the individual dog and the *D*. *caninum* infection incidence at the end of the study period was calculated for each group using the formula:

Infection incidence (%) = (No. of dogs infected in each group / No. of dogs enrolled in each group) × 100.

Significance was determined by comparing the infection incidence in each of the treated groups with the sterile water-treated control group (SAS Version 9.3 TS Level 1M2). Proportions were compared between the groups using a Fisher’s exact test. Significance of the two sided significance test was set at 5%.

## Results

All 8 dogs in the placebo treated control group were observed to be shedding *D. caninum* proglottids: there were 3 positive control dogs at 35 days after treatment, 1 positive control dog at 38 days after treatment, and 4 positive control dogs at 43 days after treatment. Transmission of *D*. *caninum* to all control dogs confirms that the challenge is adequate. No dog in either of the fluralaner-treated groups shed *D. caninum* proglottids at any time during the post-treatment observation period between 35 and 113 days post-treatment. Therefore, both oral and topically administered fluralaner were 100% effective for prevention of transmission of *D. caninum* tapeworms to dogs in this natural flea infestation model. This difference between the proportion of *D. caninum*-infected dogs in the control and treated groups was significant (Fisher’s exact test, *P* < 0.0001) (Table 2).

**Table 2:** *Dipylidium caninum* infection incidence for dogs treated and subsequently challenged with *D*. *caninum* infected *Ctenocephalides felis*

Treatment		Sterile water	Topical fluralaner	Oral fluralaner	*P-*value^a^
No. of proglottid-shedding dogs per 8 dog group		8	0	0	< 0.0001
*Dipylidium caninum* infection prevention efficacy		na	100%	100%	

^a^For each treated group compared to the placebo-treated control using Fisher’s exact test

*Abbreviation*: na - not applicable

## Discussion

These results show that treatment with either topically or orally administered fluralaner kills fleas with sufficient rapidity to prevent transmission of *D*. *caninum* to dogs throughout the 84-day period following administration of a single dose. The overall study period extended to 113 days to allow maturation of any *D*. *caninum* possibly infecting the intestinal tracts of dogs. This result is consistent with the flea control observed following oral fluralaner administration to dogs in field challenge situations [16, 21, 22] and in laboratory challenges [13, 22]. The fluralaner onset of activity following oral administration to dogs is rapid, with mortality observed at 1 hour after dosing; significant flea mortality compared with untreated control dogs at 2 hours; and 99.4% mortality of adult fleas by 8 hours of dosing [13].

The *D*. *caninum* challenge presented to dogs in this study was greater than might be encountered under natural conditions. Field survey data in Europe indicate that 5.2% of fleas infesting client-owned dogs are infected with *D*. *caninum* [7], while in this study, all dogs were experimentally infested with approximately 100 fleas at weekly intervals, with 13 to 68% of the challenge fleas estimated to be *D*. *caninum*-infected (Table 1). *Dipylidium caninum* can also be transmitted by biting lice (*Trichodectes canis*), a transmission mechanism not addressed in the present study [23] although field efficacy has been shown for fluralaner against the sucking louse, *Linognathus setosus* [24].

Flea challenges began 7 days after treatment and continued until 83 days after treatment. Housing and dog feces were examined daily for *D*. *caninum* proglottids from 35 days until 113 days (30 days after the final flea challenge) after treatment. The intervals between the initial flea challenge on day 7, the start of the observation period on day 35, the final flea challenge (day 83) and the end of the study (day 113) provided time to allow the parasite to complete its pre-patent period in any infected dog and begin shedding proglottids. No proglottids were seen in the feces of any fluralaner treated dog, regardless of the route of administration, at any time point. Detection of *D*. *caninum* proglottids in feces, cage floor and bedding is sufficiently sensitive to detect infected dogs [18].

The cysticercoid larvae of *D*. *caninum* require 24–36 hours following arrival of the flea on the dog to become infective for the definitive host. These results confirm that either oral or topical administration of fluralaner effectively kills fleas before this *D*. *caninum* development time has elapsed and is consistent with the reported speed of kill times for orally administered fluralaner over the recommended retreatment interval [13].

## Conclusions

The insecticidal efficacy of a single treatment of either orally or topically administered fluralaner prevented *D. caninum* transmission from infected fleas to susceptible dogs for up to 12 weeks following administration.

## Abbreviation

PCR: polymerase chain reaction
